# Supplementary material for: Massive yet grossly underestimated global costs of invasive insects
Source: Nat Commun. 2016 Oct 4;7:12986. doi: 10.1038/ncomms12986 (PMC5059451; doi:10.1038/ncomms12986)
Supplement: Supplementary Information — Supplementary Figures 1 - 9, Supplementary Table 1, Supplementary Note 1, Supplementary Methods and Supplementary References [file ncomms12986-s1.pdf]

Supplementary Figures

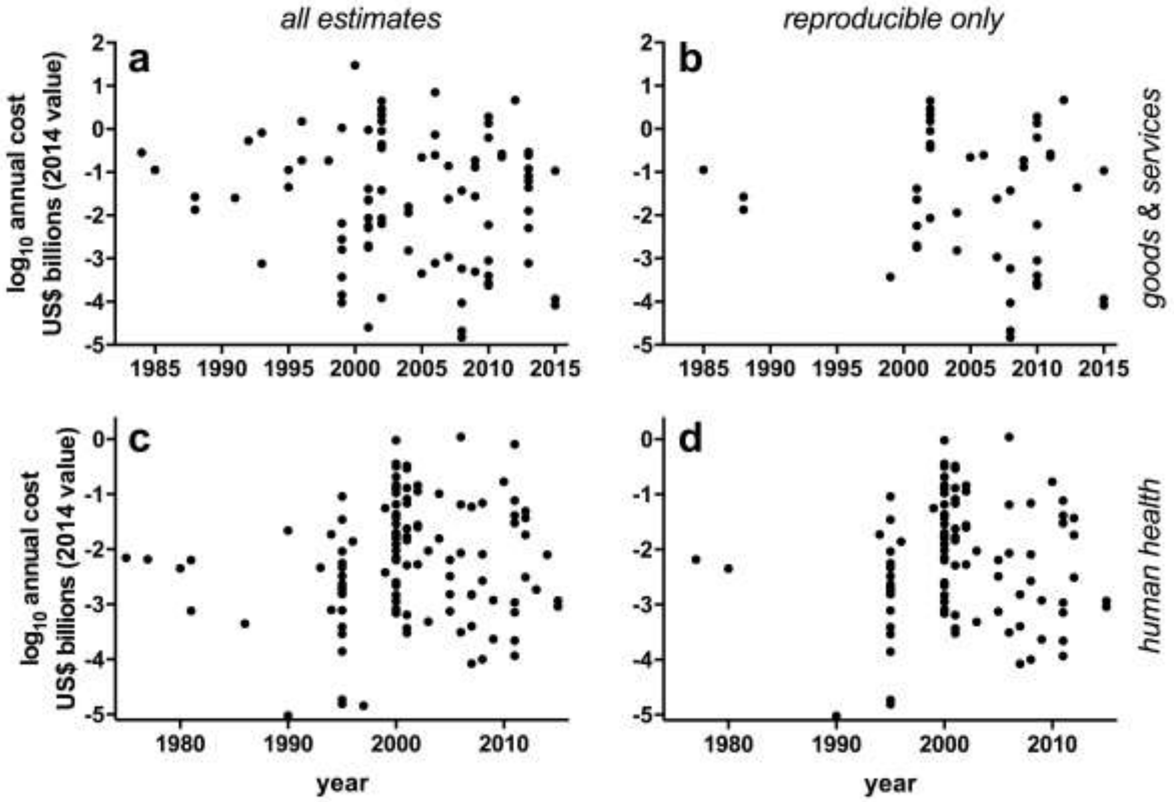

Supplementary Figure 1 | No relationship between annual cost rate and year of applicability for goods and services estimates (**a**, **b**) and human health estimates (**c**, **d**). The left-hand panels (**a**, **c**) include all estimates regardless of reproducibility, whereas the right-hand panels (**b**, **d**) only include costs for which estimates can be verified ('reproducible'). All costs expressed as annual 2014 US dollars.

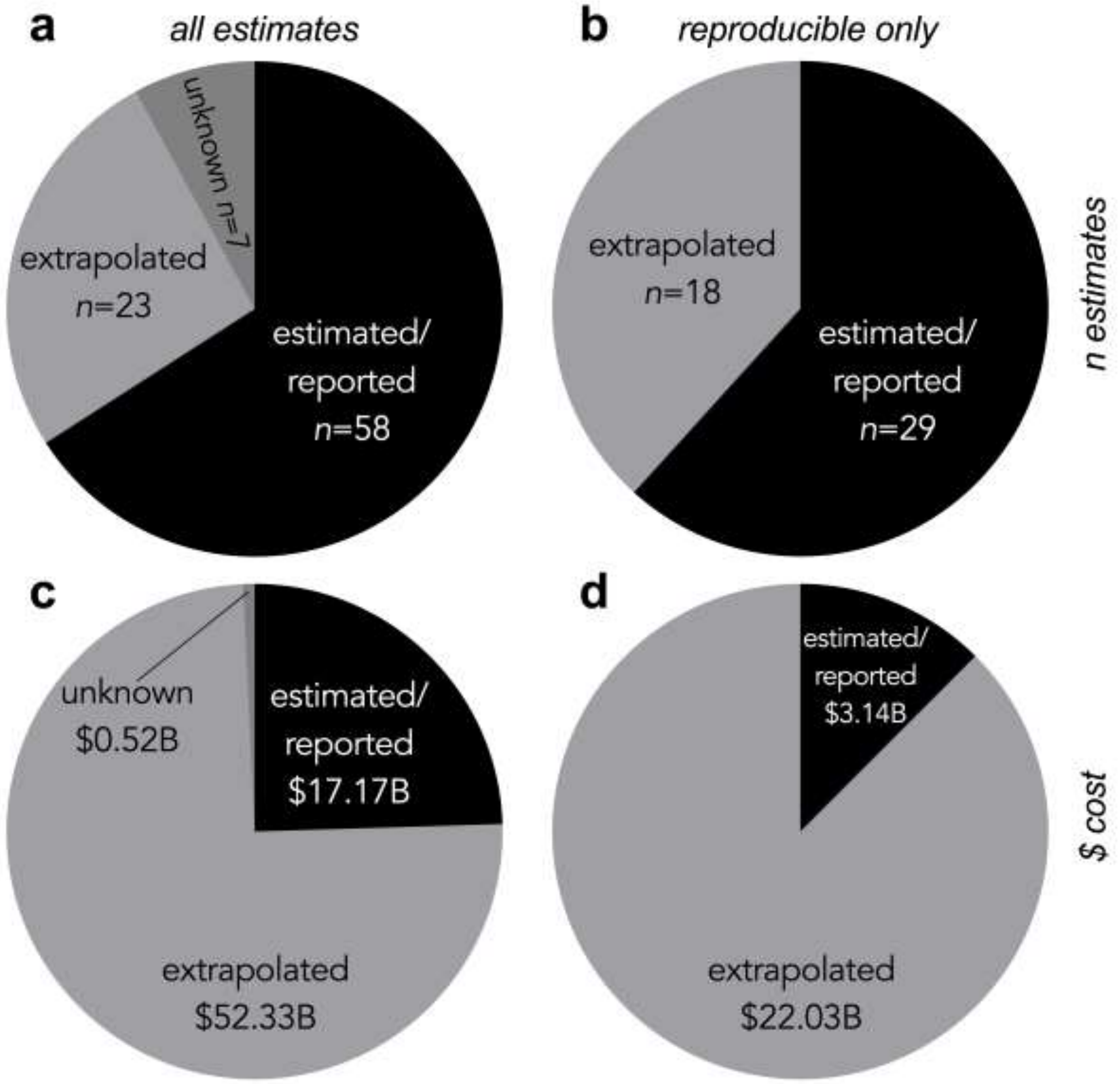

Supplementary Figure 2 | Summary of goods and services costs due to invasive insects by expenditure type (i.e., *measured/reported estimates, modelled extrapolations, unknown*). The top panels (**a**, **b**) indicate the number of estimates examined per category, whereas the bottom panels (**c**, **d**) summarise by total cost (2014 US$). The left-hand panels (**a, c**) include all estimates regardless of reproducibility, whereas the right-hand panels (**b**, **d**) only include costs for which estimates can be verified ('reproducible').

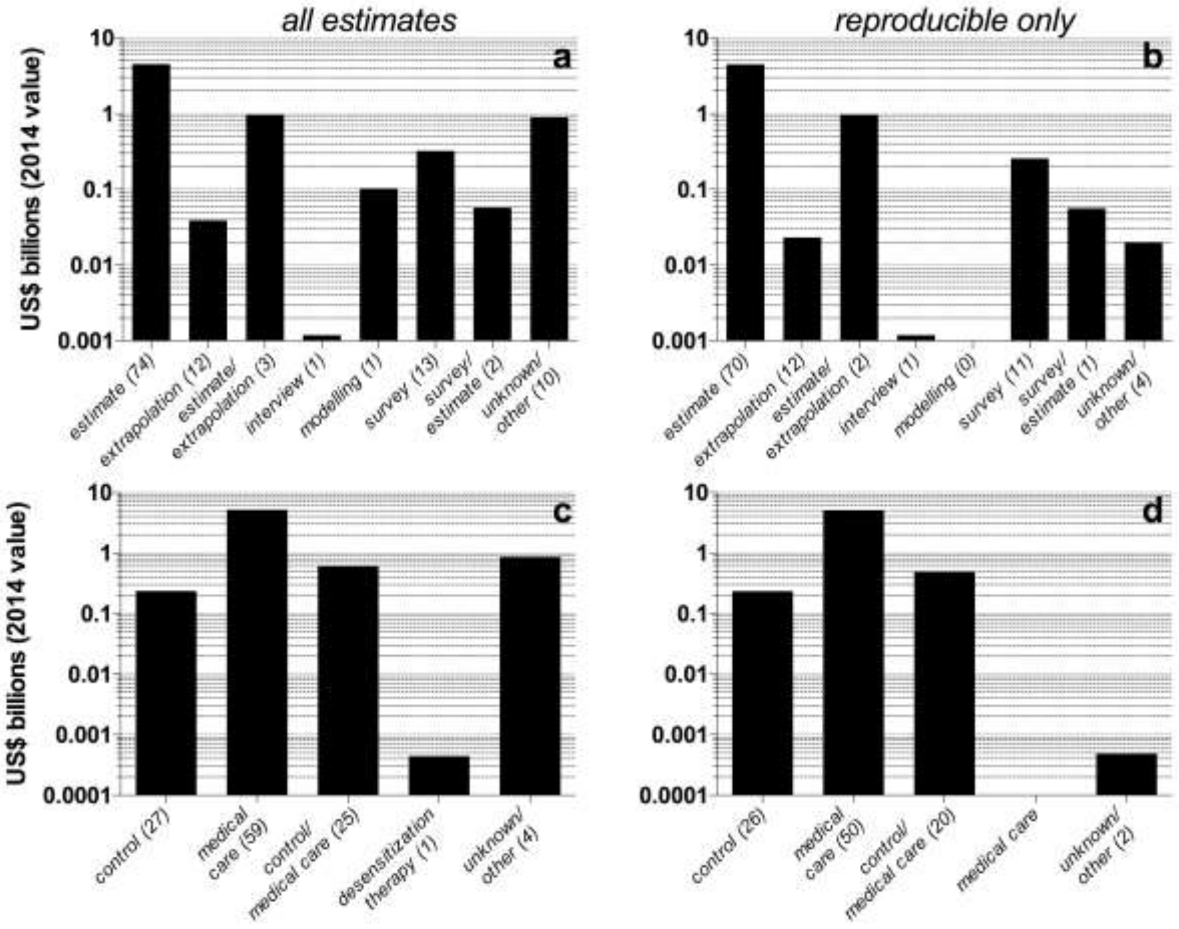

Supplementary Figure 3 | Summary of human health costs due to invasive insects by expenditure type (**a**, **b**) and expenditure target (**c**, **d**). The left-hand panels (**a**, **c**) include all estimates regardless of reproducibility, whereas the right-hand panels (**b**, **d**) only include costs for which estimates can be verified ('reproducible'). All costs expressed as annual 2014 US dollars.

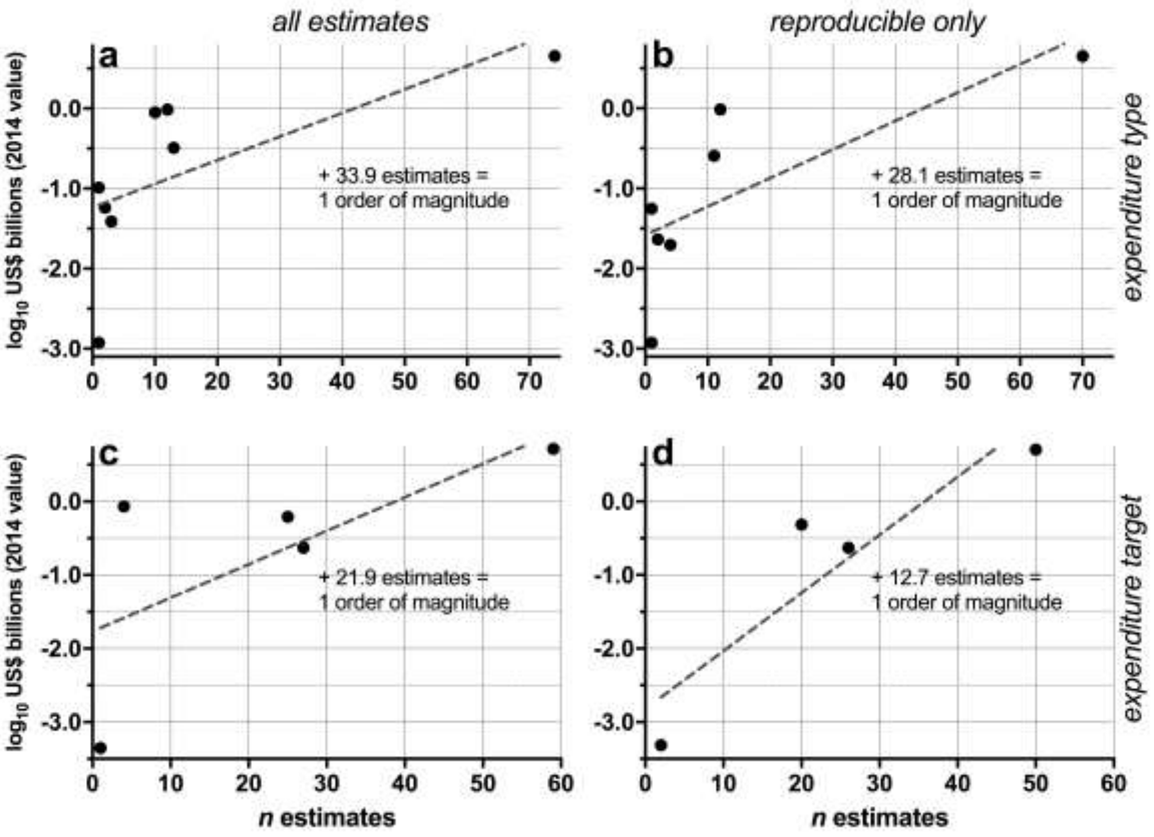

Supplementary Figure 4 | Human health costs due to invasive insects by expenditure type (a, b) and expenditure target (c, d) relative to the number of estimates made within each category. The left-hand panels (a, c) include all estimates regardless of reproducibility, whereas the right-hand panels (b, d) only include costs for which estimates can be verified ('reproducible'). All costs expressed as annual 2014 US dollars.

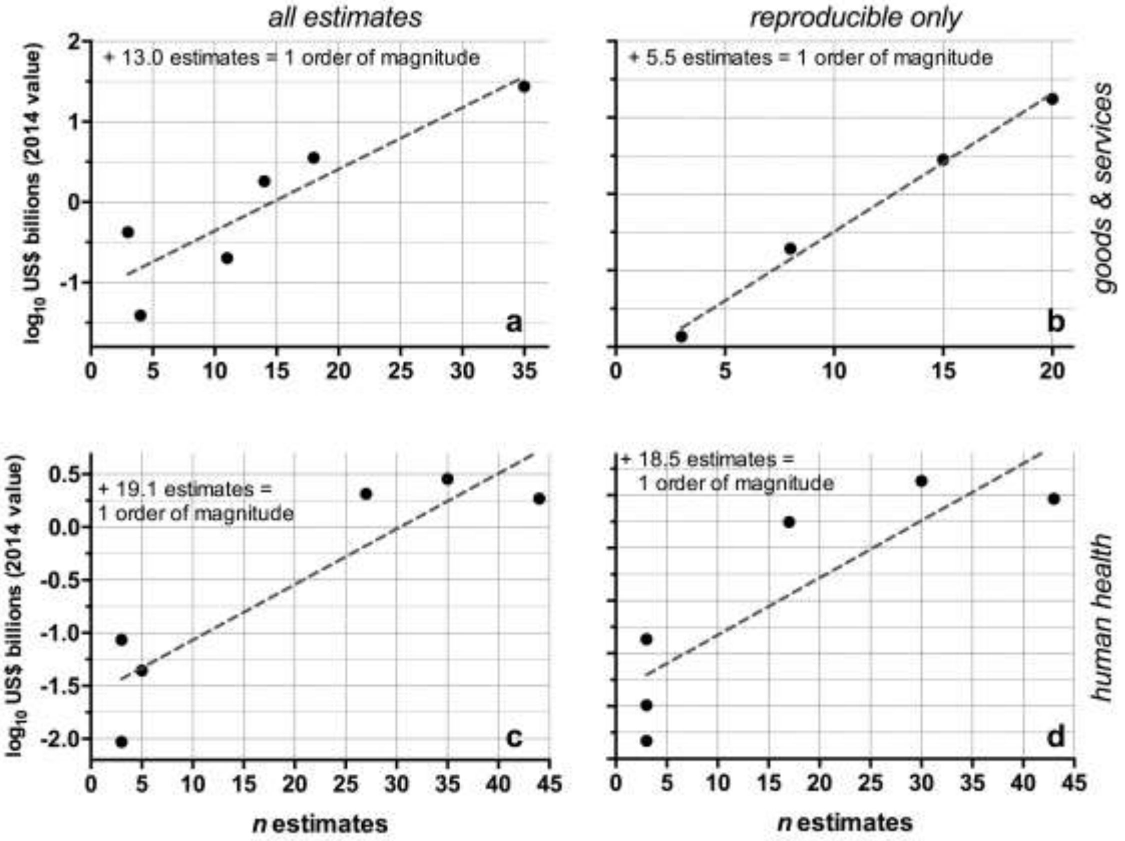

Supplementary Figure 5 | Sums of costs per region (regional aggregations from Fig. 2a,b and 3a,b) due to invasive insects relative to the number of estimates made for goods and services (**a**, **b**) and human health (**c**, **d**). The first column includes all estimates regardless of reproducibility (**a**, **c**) whereas the second only includes costs for which estimates can be verified ('reproducible') (**b**, **d**). All costs expressed as annual 2014 US dollars.

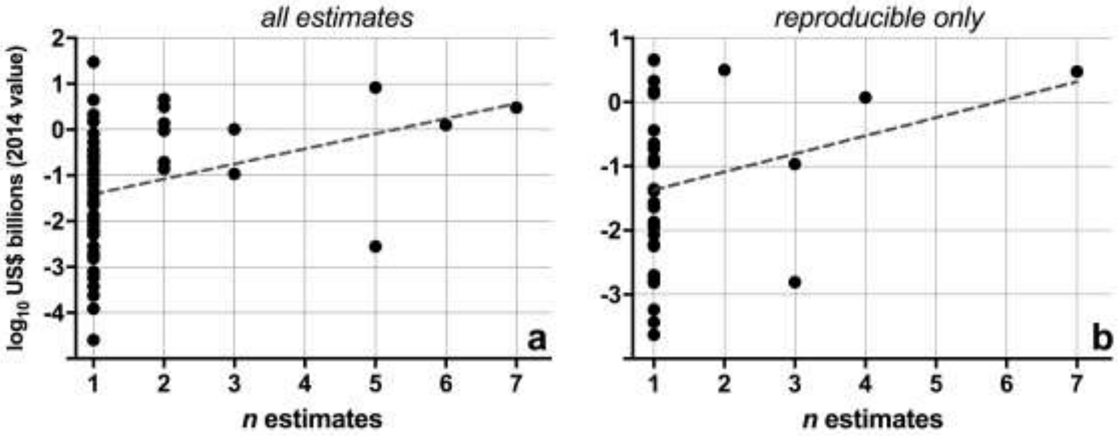

Supplementary Figure 6 | By-species sums of costs due to invasive insects relative to number of estimates made for goods and services. The left-hand panel (**a**) includes all estimates regardless of reproducibility, whereas the right-hand panel (**b**) only includes costs for which estimates can be verified ('reproducible'). All costs expressed as annual 2014 US dollars.

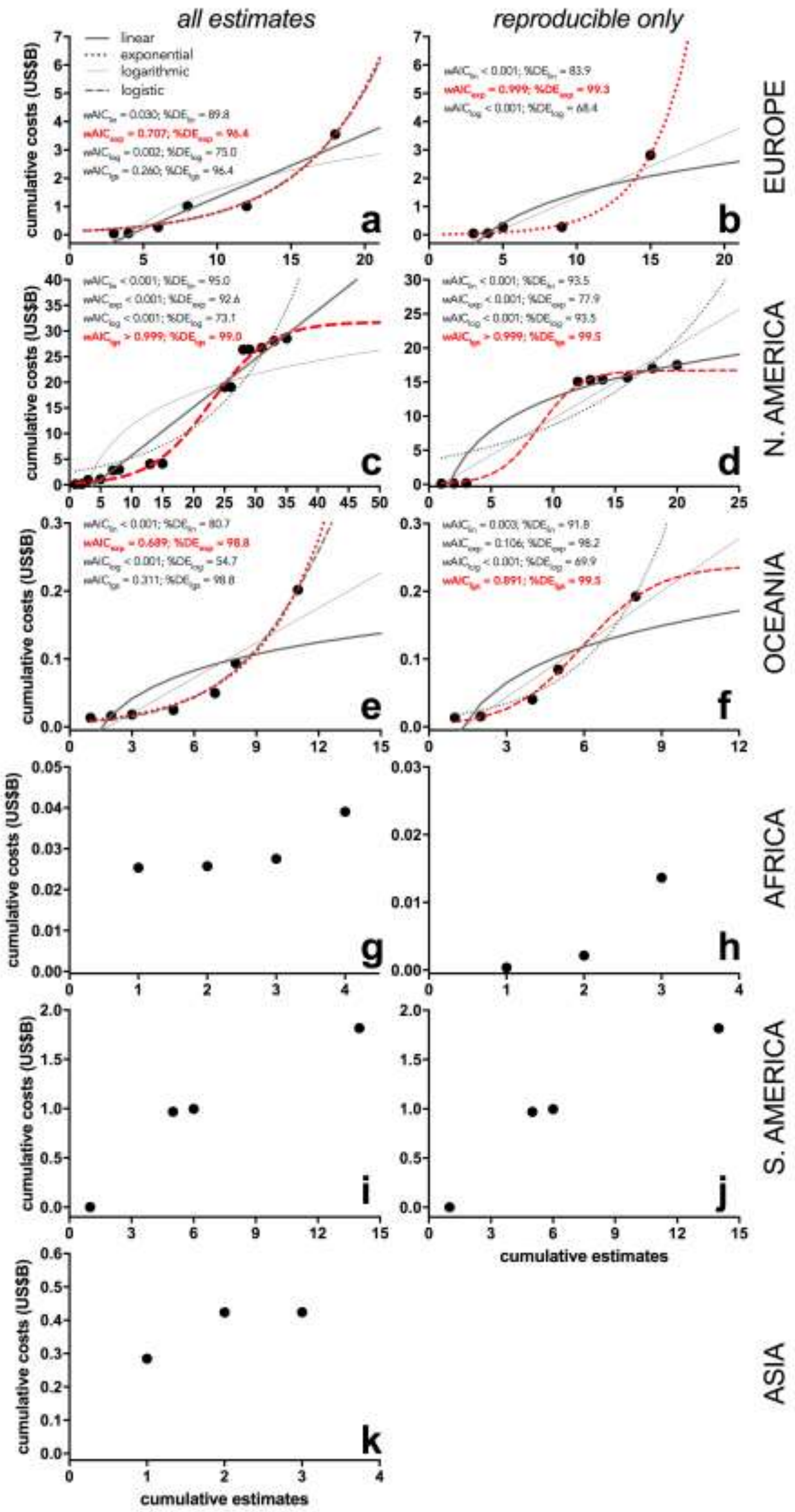

Supplementary Figure 7 | Regional cumulative costs due to invasive insects relative to the number of estimates for goods and services costs for all estimates (**a**, **c**, **e**, **g**, **i**, **k**) and reproducible-only estimates (**b**, **d**, **f**, **h**, **j**). For a given year t , we summed all values (costs and number of estimates) up to *t*. We fitted linear (lin), exponential (exp),

logarithmic (log) and logistic (lgs) models to each curve to examine evidence for asymptotic behaviour (identified by the dominance of a logarithmic or logistic model). The top-ranked model in each case is indicated in red (fitted line and model performance characteristics – see also 'Sampling bias' in the Supplementary Methods). Models could be fitted to estimates for Europe (**a**, **b**), North America (**c**, **d**), and Oceania (**e**, **f**) only (the logistic model for reproducible-only estimates in Europe failed to converge). Shown are the Akaike's information criterion (AIC) weights (*w*AIC ≈ relative model probability) and explained deviance in the data (%DE ≈ coefficient of determination) for each model and region/category. All costs expressed as 2014 US dollars.

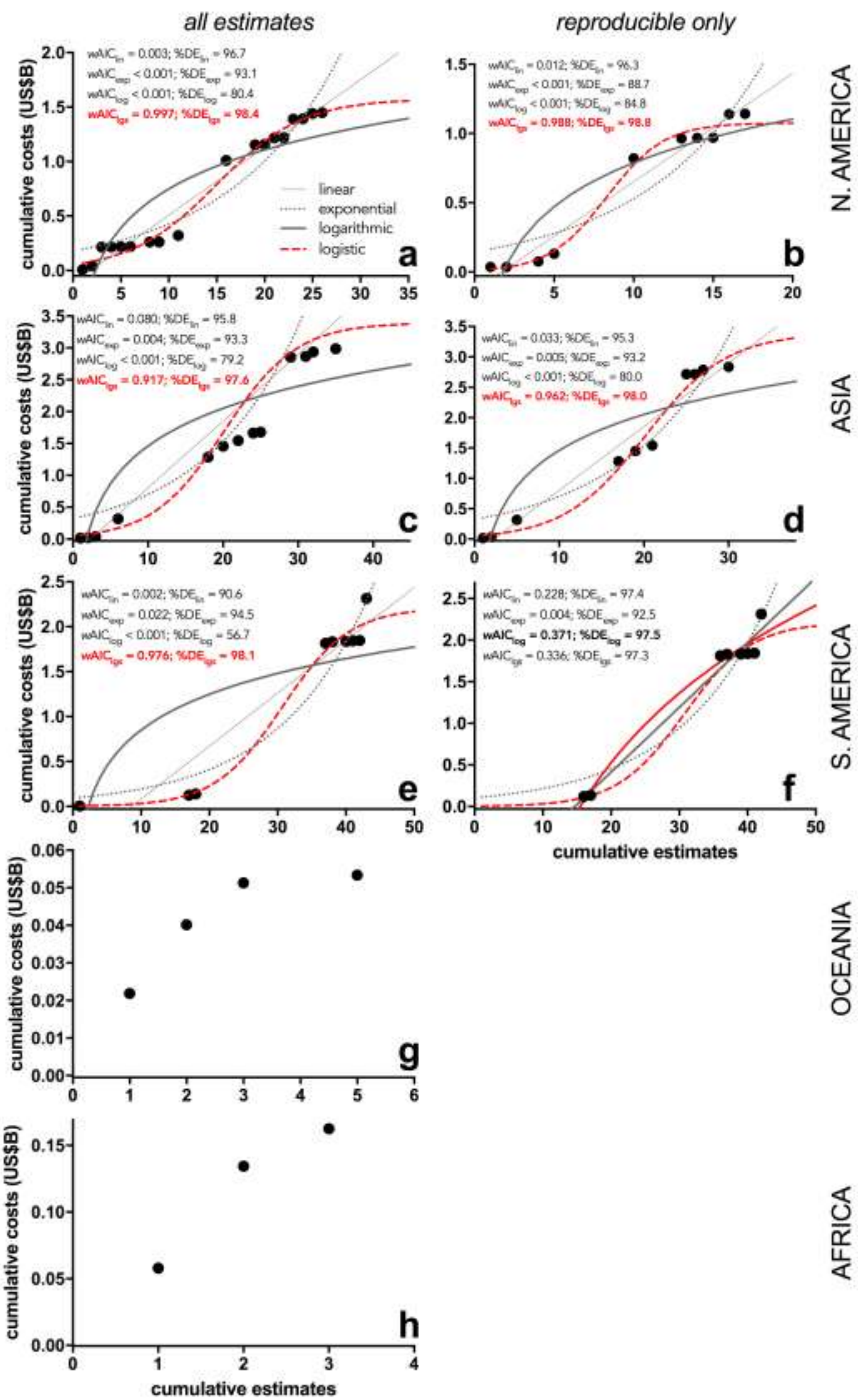

Supplementary Figure 8 | Regional cumulative costs due to invasive insects relative to the number of estimates for human health costs for all estimates (**a**, **c**, **e**, **g**, **h**) and reproducible-only estimates (**b**, **d**, **f**)**.** For a given year t , we summed all values (costs

and number of estimates) up to *t*. We fitted linear (lin), exponential (exp), logarithmic (log) and logistic (lgs) models to each curve to examine evidence for asymptotic behaviour (identified by the dominance of a logarithmic or logistic model). The top-ranked model in each case is indicated in red (fitted line and model performance characteristics – see also 'Sampling bias' in the Supplementary Methods). Models could be fitted to estimates for North America (**a**, **b**), Asia (**c**, **d**), and South America (**e**, **f**) only. Shown are the Akaike's information criterion (AIC) weights (*w*AIC ≈ relative model probability) and explained deviance in the data (%DE ≈ coefficient of determination) for each model and region/category. All costs expressed as 2014 US dollars.

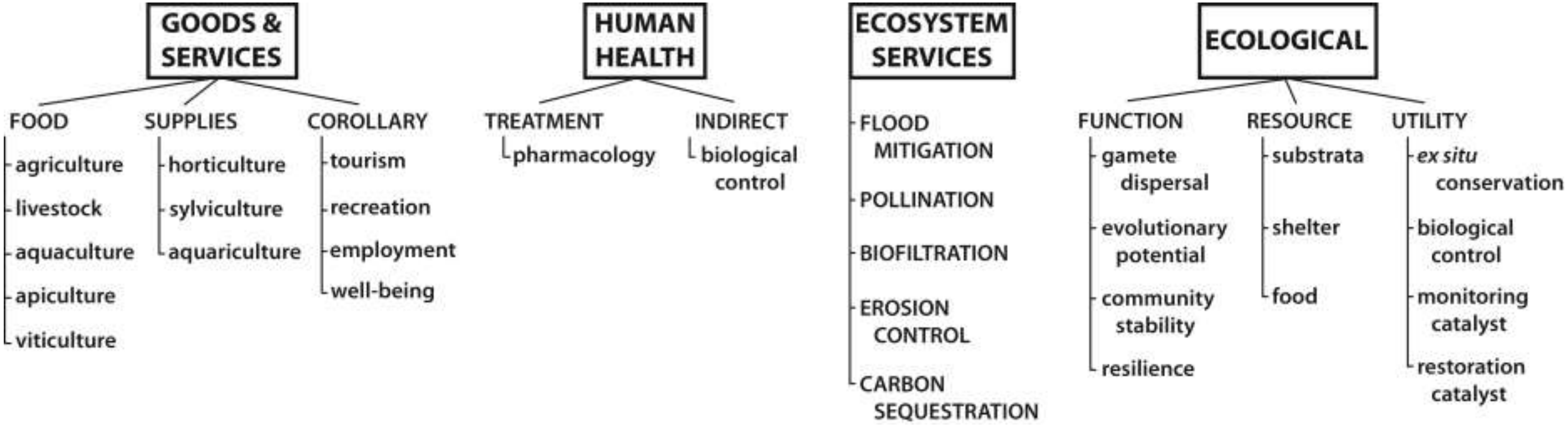

Supplementary Figure 9 | Benefit (potential and observed) categories associated with invasive species.

Supplementary Tables

Supplementary Table 1 | Model fits to cumulative cost curves.

MODEL	goods & services (all)	goods & services (reproducible)	human health (all)	human health (reproducible)
<u>*w*AIC</u>				
linear	< 0.001	0.650	< 0.001	< 0.001
exponential	< 0.001	0.001	< 0.001	< 0.001
logarithmic	< 0.001	0.003	< 0.001	< 0.001
logistic	0.999	0.346	> 0.999	> 0.999
<u>%DE</u>				
linear	93.1	91.0	97.3	97.2
exponential	78.7	80.1	93.3	92.3
logarithmic	82.4	82.1	73.1	72.5
logistic	96.5	91.5	99.5	99.2

Akaike's information criterion weights (*w*AIC) and percentage deviance explained (%DE) for four models fitted to cumulative costs and estimates curves for goods and services and human health costs arising from invasive insects (for both 'all estimates' and 'reproducible-only estimates' separately).

Supplementary Notes

Supplementary Note 1

Direct *versus* indirect health costs. The economic burden of dengue and chikungunya has three components: (1) illness, (2) surveillance and control, and (3) indirect costs. However, few studies have dealt with or attempted to evaluate indirect costs. Health and wealth are closely linked, with life expectancy related to wealth, vector-borne diseases related to poverty, and dengue epidemics most likely to arise and persist under conditions commonly created as a result of low income[1-3]. Recent chikungunya epidemics in India highlight the impact these have on people from low socioeconomic backgrounds[4] as 80% of those affected were below the poverty line of US$1 person^{-1} day^{-1}. Poverty is therefore an important determinant of chikungunya infection. Chikungunya infection exacerbates the problems of poverty because it entails additional health-care expenditure and loss of income due to lowered productivity, thereby reinforcing the poverty-ill-health nexus[5]. Other ways in which the disease affects the economy are through a reduction in productivity at work (due to fatigue, weariness, absence and the care of family members and associates) and loss of investments. It has

been estimated that the 2005-2006 chikungunya epidemic in La Réunion resulted in a loss of just under one economy growth point (0.9) in that year[6,7].

As well as decreasing the productivity of affected populations, epidemics are likely to impact tourism heavily, a mainstay of many economies. For example, as a result of the serious outbreak of chikungunya in La Réunion in 2005 and 2006, which affected about 300,000 people, tourism on the island dropped by 30% in 2006, with the loss of 500 salaried jobs[7]. The estimated costs of chikungunya in terms of a 4% decline in tourists from non-endemic countries resulted in a substantial loss of tourism revenues of at least US$8 million for Gujarat, US$65 million for Malaysia and US$363 million for Thailand[8]. The estimated immediate annual cost of chikungunya and dengue to these economies is approximately US$90 million, US$133 million and US$127 million, respectively[8]. Another indirect cost related to control is personal protection. In La Réunion, household expenditure for protection against *Aedes* spp. mosquitoes over a one-year period has been extrapolated to US$28.05 million (range: US$25.58—30.76 million)[9]. In India, the annual expenditure on personal protection measures in urban areas amounts to 0.63% of per-capita income[10].

Other indirect effects of epidemics include the impact on the blood-supply system, as revealed by studies of the chikungunya epidemic in Italy[11] and the West Nile virus epidemic in the USA[12], and the cost of vaccination when available, as for yellow fever[13]. Finally, we did not account for the impact on quality of life. For example, it has been estimated that the tiger mosquito *Aedes albopictus* prevents 59.5% of residents from enjoying outdoor activities at least to some extent[14]. Additionally, these indirect (and direct) costs could be much larger considering the current epidemic of the *Aedes*-borne zika virus (that has caused around 5000 microcephaly cases in Brazil), with direct medical cost of $91,102 per microcephaly case and lifetime[15], and with huge indirect costs on tourism, personal protection, etc. not yet estimated.

For the studies we examined, none estimated only indirect costs; in fact, 78.8% of all studies and 92.7% of the reproducible-only studies included both direct and indirect costs that could not be separated.

Supplementary Methods

Expenditure types and targets. We categorized goods and services costs according to their expenditure type (i.e., *estimates, extrapolations, unknown*) to determine to what extent summaries relied on projected *versus* measured elements (Supplementary Fig. 2). Even though most studies were measured estimates (all studies: 65.9%; reproducible-only studies: 61.7%), the sum of the total costs were dominated by modelled extrapolations (all studies: 74.7%; reproducible-only studies: 87.5%) (Supplementary Fig. 2).

We also categorized health costs according to their expenditure type (i.e., estimates, extrapolation, estimate+extrapolation, interview, modelling, survey, survey+estimate, unknown) and expenditure target (i.e., control, medical care, control+medical care, desensitization therapy, unknown) (Supplementary Fig. 3). In contrast to the goods and services categorization, this shows clearly that most health

care costs are estimates (65.5% for all studies; 77.3% for reproducible-only studies) targeting medical care (75.2% for all studies; 87.6% for reproducible-only studies). However, there is still the sampling problem identified with the regional sums — total amounts per category tend to increase with an increasing sampling effort (i.e., number of studies) (Supplementary Fig. 4). For expenditure type, most studies [74/116] are predictably 'estimates', mostly (59/116) targeting medical care.

Sampling bias. Plotting cost sums against the number of estimates available reveals a potentially strong sampling bias (i.e., the more studies done and cost estimates made, the higher the total estimated costs). Expressing this as regional sums for both goods and services and human health costs reveals a strong, positive relationship (Supplementary Fig. 5)

We also expressed global cumulative costs due to invasive insects relative to the number of cumulative estimates for goods and services- and human health-related costs, and for all estimates and reproducible-only estimates. We summed the cumulative values for year t up to t globally and displayed the relationship between cumulative costs and the associated number of cumulative estimates (Fig. 4). There were clear step changes in the cost estimates driven by the occasional high-cost estimate (Fig. 4).

To examine whether the cumulative costs were approaching some sort of asymptote that might indicate a slowing in the acceleration of estimated damages, we fit four simple functions to the cumulative curves: (*i*) linear: $y = \beta_0 + \beta_1 x$ (where y = cost, x = number of estimates, and β_0, β_1, … are fitted coefficients); (*ii*) exponential: $y = \beta_0(\beta_1)^x$; (*iii*) logarithmic: $y = \beta_0 + \beta_1 \log_e x$; and (*iv*) logistic: $y = \beta_0/\left(1 + \beta_1 e^{-\beta_2 x}\right)$. For each category (goods and services; human health) and type (all; reproducible-only), we calculated Akaike's information criterion (AIC = log-likelihood + 2k, where k = number of model parameters) weights (wAIC — relative AIC standardised to sum to 1 across all models considered) as an index of relative model probability[16,17], and the percentage deviance explained (%DE) as an index of goodness-of-fit (analogous to a least-squares R^2 value) for each model. Our hypothesis was that if the costs demonstrated a slowing in the acceleration of cumulative values, the logarithmic or logistic models would have more support and explain more of the deviance in the data.

In most cases (reproducible-only goods and services costs being the exception), the logistic was clearly the most supported model for the global cumulative curves (Supplementary Table 1).

For reproducible-only goods and services costs, however, the linear model had the most support (wAIC = 0.650), indicating only weak evidence for an asymptotic-cost threshold. This result, combined with the large difference between the 'all' and 'reproducible-only' goods and services estimates, suggests that global goods and services costs in particular are grossly underestimated due to under-sampling, and that globally we are still in the initial phases of total cost estimation.

It is important to note that the evidence for asymptotic cumulative costs does not necessarily imply that all costs have been adequately measured; rather, it only indicates

that the costliest components of most major invasive insects have been estimated first, followed by lower costs associated with new areas becoming invaded or new insect pests being introduced to novel areas. The series of plateaus we discovered in many of the better-sampled series also indicates that there are step changes in the cumulative costs, and that future plateaus beyond the upper ones identified are still likely.

We also applied the four-model fitting procedure to the accumulated costs within each region (for regions with adequate sampling to fit the models – see below). For goods and services costs, we again fitted the linear, exponential, logarithmic and logistic models to estimates from Europe, North America, and Oceania (Supplementary Fig. 7); for human health costs, we fitted the models to estimates from North America, Asia, and South America (Supplementary Fig. 8) (all other regions had ≤ 4 cumulative estimates for which deemed model fitting statistically dubious).

Supplementary References

1 Bloom, D. & Canning, D. Epidemics and Economics. Program on the Global Demography of Aging. PGDA Working Paper No. 9. (Harvard School of Public Health, Boston, 2006).

2 Bonds, M. H., Dobson, A. P. & Keenan, D. C. Disease ecology, biodiversity, and the latitudinal gradient in income *PLoS Biol.* **10**, e1001456, doi:10.1371/journal.pbio.1001456 (2012).

3 Roser, M. *Life Expectancy*, <ourworldindata.org/data/population-growth-vital-statistics/life-expectancy> (2016).

4 Kumar, C. J. *et al.* The socioeconomic impact of the chikungunya viral epidemic in India *Open Medicine* **1**, www.openmedicine.ca/article/view/143/189 (2007).

5 Gopalan, S. S. & Das, A. Household economic impact of an emerging disease in terms of catastrophic out-of-pocket health care expenditure and loss of productivity: investigation of an outbreak of chikungunya in Orissa, India *J. Vector-Borne Dis.* **46**, 57-64 (2009).

6 Fontenille, D. *et al.* La lutte antivectorielle en France. (IRD Éditions, Marseille, France, 2009).

7 Institut national de la statistique et des etudes economiques. Economic Balance Assessment 2006: Synthesis, Chikungunya, consumption-income. Revue Économie de la Réunion 2. (2007).

8 Mavalankar, D., Puwar, T., Murtola, T. M. & Vasan, S. S. Quantifying the Impact of Chikungunya and Dengue on Tourism Revenues. Working paper no. 2009-02-03. (Indian Institute of Management, Ahmedabad, India, 2009).

9 Thuilliez, J., Bellia, C., Dehecq, J.-S. & Reilhes, O. Household-level expenditure on protective measures against mosquitoes on the island of La Réunion, France *PLoS Negl. Trop. Dis.* **8**, e2609, doi:10.1371/journal.pntd.0002609 (2014).

10 Snehalatha, K. S., Ramaiah, K. D., Vijay Kumar, K. N. & Das, P. K. The mosquito problem and type and costs of personal protection measures used in rural and urban communities in Pondicherry region, South India *Acta. Trop.* **88**, 3-9 (2003).

11 Liumbruno, G. M. *et al.* The chikungunya epidemic in Italy and its repercussion on the blood system *Blood Transfus* **6**, 199-210 (2008).

12 Lee, B. Y. & Biggerstaff, B. J. Screening the United States blood supply for West Nile virus: a question of blood, dollars, and sense *PLoS Med.* **3**, e99, doi:10.1371/journal.pmed.0030099 (2006).

13 Zengbe-Acray, P. *et al.* Estimated operational costs of vaccination campaign to combat yellow fever in Abidjan *Santé Publique* **21**, 383-391 (2009).

14 Halasa, Y. A. *et al.* Quantifying the impact of mosquitoes on quality of life and enjoyment of yard and porch activities in New Jersey *PLoS ONE* **9**, e89221, doi:10.1371/journal.pone.0089221 (2014).

15 Alfaro-Murillo, J. A. *et al.* A cost-effectiveness tool for informing policies on zika virus control *PLoS Negl. Trop. Dis.* **10**, e0004743, doi:10.1371/journal.pntd.0004743 (2016).

16 Burnham, K. P. & Anderson, D. R. *Model Selection and Multimodel Inference: A Practical Information-Theoretic Approach*. 2nd edn, (Springer-Verlag, 2002).

17 Burnham, K. P. & Anderson, D. R. Understanding AIC and BIC in model selection *Sociological Methods and Research* **33**, 261-304 (2004).
